# End-tidal carbon dioxide monitoring using a naso-buccal sensor is not appropriate to monitor capnia during non-invasive ventilation

**DOI:** 10.1186/s13613-014-0042-8

**Published:** 2015-02-12

**Authors:** Lise Piquilloud, David Thevoz, Philippe Jolliet, Jean-Pierre Revelly

**Affiliations:** Adult Intensive Care and Burn Unit, University Hospital of Lausanne, Lausanne, Switzerland; Cardio-Respiratory Physiotherapy Unit, University Hospital of Lausanne, Lausanne, Switzerland

**Keywords:** Respiratory monitoring, Non-invasive ventilation, End-tidal CO_2_, Hypercapnic respiratory failure

## Abstract

**Background:**

In acute respiratory failure, arterial blood gas analysis (ABG) is used to diagnose hypercapnia. Once non-invasive ventilation (NIV) is initiated, ABG should at least be repeated within 1 h to assess PaCO_2_ response to treatment in order to help detect NIV failure. The main aim of this study was to assess whether measuring end-tidal CO_2_ (EtCO_2_) with a dedicated naso-buccal sensor during NIV could predict PaCO_2_ variation and/or PaCO_2_ absolute values. The additional aim was to assess whether active or passive prolonged expiratory maneuvers could improve the agreement between expiratory CO_2_ and PaCO_2_.

**Methods:**

This is a prospective study in adult patients suffering from acute hypercapnic respiratory failure (PaCO_2_ ≥ 45 mmHg) treated with NIV. EtCO_2_ and expiratory CO_2_ values during active and passive expiratory maneuvers were measured using a dedicated naso-buccal sensor and compared to concomitant PaCO_2_ values. The agreement between two consecutive values of EtCO_2_ (delta EtCO_2_) and two consecutive values of PaCO_2_ (delta PaCO_2_) and between PaCO_2_ and concomitant expiratory CO_2_ values was assessed using the Bland and Altman method adjusted for the effects of repeated measurements.

**Results:**

Fifty-four datasets from a population of 11 patients (8 COPD and 3 non-COPD patients), were included in the analysis. PaCO_2_ values ranged from 39 to 80 mmHg, and EtCO_2_ from 12 to 68 mmHg. In the observed agreement between delta EtCO_2_ and deltaPaCO_2_, bias was −0.3 mmHg, and limits of agreement were −17.8 and 17.2 mmHg. In agreement between PaCO_2_ and EtCO_2_, bias was 14.7 mmHg, and limits of agreement were −6.6 and 36.1 mmHg. Adding active and passive expiration maneuvers did not improve PaCO_2_ prediction.

**Conclusions:**

During NIV delivered for acute hypercapnic respiratory failure, measuring EtCO_2_ using a dedicating naso-buccal sensor was inaccurate to predict both PaCO_2_ and PaCO_2_ variations over time. Active and passive expiration maneuvers did not improve PaCO_2_ prediction.

**Trial registration:**

ClinicalTrials.gov: NCT01489150.

## Background

Non-invasive ventilation (NIV) is widely used [1] in emergency rooms, in intensive and intermediate care units, and in recovery rooms to treat de novo and, even if it is more debatable [2,3], postextubation hypercapnic respiratory failure. Arterial blood gas analysis (ABG) is usually performed to diagnose hypercapnia and should at least be repeated within 1 h after NIV initiation to assess PaCO_2_ response to treatment [1]. However, as follow-up ABG requires a new arterial puncture in patients not previously equipped with an arterial line, this exam is often postponed with the risk of delaying NIV failure diagnosis and intubation, a condition previously associated with poor outcome [4]. Only a reliable non-invasive monitoring of the course of PaCO_2_ during NIV could avoid such a delay and help optimizing ventilator settings. End-tidal CO_2_ (EtCO_2_) monitoring is easy to perform and widely used during anesthesia to assess the adequacy of delivered minute ventilation without performing repetitive ABG [5,6]. Using capnometry to monitor capnia in non-intubated patients during NIV is much more challenging. Indeed, during NIV, gas leak occurs in the respiratory ‘circuit’ and conceivably, in this situation, only gas sampling directly at the level of the patient’s airways can reflect true expiratory gas.

As new specialized naso-buccal EtCO_2_ sensors have recently been developed to collect expired gas directly at the airway opening, there is now an opportunity to use capnometry to monitor capnia during NIV. The main aim of this study was to assess the ability of a dedicated EtCO_2_ naso-buccal sensor to predict PaCO_2_ variations and/or PaCO_2_ absolute values in hypercapnic patients during NIV. The second aim of the study was to assess whether active or passive prolonged expiratory maneuvers could improve the agreement between expiratory CO_2_ and PaCO_2_.

## Methods

A prospective pilot study was conducted in our medico-surgical ICU in Lausanne, Switzerland. The hospital ethics committee (Human Research Ethics Committee of Lausanne, Switzerland) approved the study protocol, and written informed consent was obtained before inclusion in the study. In the absence of published data reporting the use of a naso-buccal sensor to measure EtCO_2_ in acutely ill patients undergoing NIV, no power computation could be performed.

### Patients

Non-intubated patients suffering from hypercapnic (PaCO_2_ ≥ 45 mmHg) acute respiratory failure, hospitalized in the ICU, equipped with an arterial line and requiring NIV could be included in the study if they had no major hemodynamic instability, no facial lesion preventing the use of the naso-buccal sensor, and no cognitive disability or psychiatric disease liable to interfere with NIV. To note, as only patients admitted in the ICU and already equipped with an arterial line could be included in the study, the NIV treatment monitored in the study was usually not the first NIV treatment delivered to the patients.

### Study protocol and measurements

**Figure 1:**
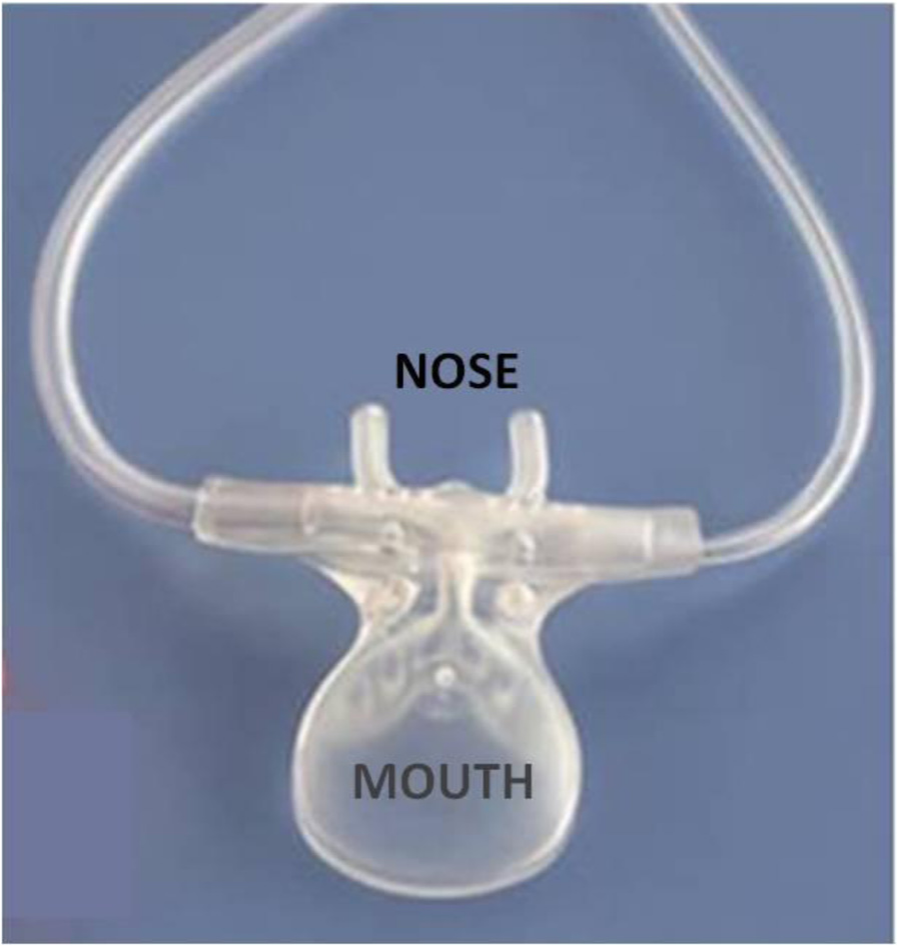
**Naso-buccal sensor.** Illustration of the naso-buccal sensor used in this study. This device is designed to collect expiratory gas immediately at the airway opening both at the nose and mouth levels.

Upon inclusion, the patient was equipped with the Smart CapnoLine® naso-buccal sensor (Figure 1) designed to collect expiratory gas immediately at the airway opening both at the nose and mouth levels connected to the Capnostream 20 monitor® (Oridion Medical Ltd, Jerusalem, Israël). To perform the measurement, a sample of gas is transmitted from the patient to a micro-cell of 15 μl located in the monitor (sidestream capnography system). A sample of gas of 50 ml/min is needed for the measurement. The measurement is performed by non-dispersive infrared spectroscopy. For each respiratory cycle, the capnogram is displayed on the Capnostream 20 monitor®. For each EtCO_2_ value recorded, the investigator checked the good quality of the capnogram displayed on the screen. The value of one respiratory cycle was recorded at each measurement time.

**Figure 2:**
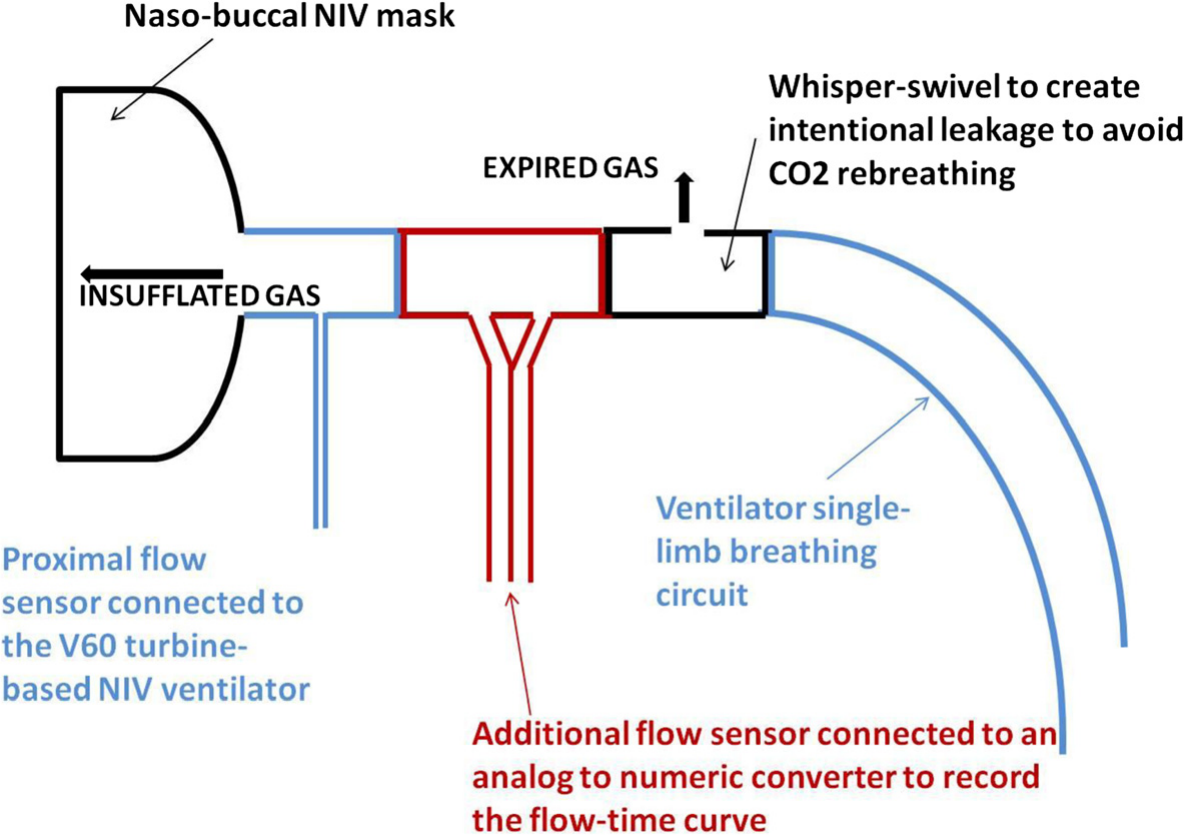
**Respiratory circuit.** Illustration of the respiratory circuit used in the study. From the patient to the ventilator, the circuit consists of an hermetic nasobuccal mask, the dedicated proximal flow sensor of the V60 ventilator, the additional flow sensor inserted to record insufflated and exuflated flow-time curves, the dedicated whisper swivel to create a calibrated intentional leak to avoid CO_2_ rebreathing, and the ventilator single-limb pipe.

ABG and the corresponding EtCO_2_ value displayed by the monitor were recorded as baseline values. NIV treatment was then initiated using an hermetic naso-buccal mask (Vygon Large®, Ecouen, France) held in place using a dedicated strap and a single-limb NIV ventilator (V60®, Respironics Philips, Amsterdam, Netherland). Calibrated intentional leakage to allow CO_2_ expiration was created in the respiratory circuit using the dedicated whisper swivel (Whisper swivel®, Respironics Philips, Amsterdam, Netherland). A flow sensor (Hamilton, Bonaduz, Switzerland) was placed between the patient and the whisper swivel and connected to an analog-to-digital converter (MP100, Biopac, Systems, Goleta, CA, USA) to continuously record the flow-time curve. The respiratory circuit with the additional flow sensor is schematized in Figure 2. ABG and EtCO_2_ values were recorded at 15, 30, 45, and 60 min after the initiation of NIV. At times corresponding to each PaCO_2_ and EtCO_2_ measurements, insufflated volumes were measured offline for ten consecutive respiratory cycles (by integration of the inspiratory flow-time curve recorded by the flow sensor placed between the patient and the whisper swivel) and the mean value was computed. Respiratory rate and delivered minute ventilation were also computed.

At 30 and 60 min after the beginning of NIV, the patient performed upon request a voluntary slow and maximal expiration. In brief, the patients were asked to slowly empty their lungs as much and for as long as possible. The expired CO_2_ value displayed at the end of this active expiration maneuver was recorded. A passive expiratory maneuver was then performed with the help of an experienced respiratory therapist (bilateral chest compression during slow expiration), and the corresponding expired CO_2_ value was recorded. The naso-buccal mask was not removed during the maximal expiratory maneuvers meaning that the patient expired through the nasobuccal sensor and the ventilator circuit and thus against the set PEEP. The backup safety respiratory frequency of the ventilator was set at 6 by minute to allow expiratory maneuvers of 10 s.

### Calculations and statistics

To assess PaCO_2_ variations, the differences between two consecutive PaCO_2_ (delta PaCO_2_) values were computed for each patient between the initial value and the 15-min value, between the 15- and 30-min values, between the 30- and 45-min values, and finally between the 45- and 60-min values. Delta EtCO_2_ were computed to assess EtCO_2_ variations according to the same procedure.

The PaCO_2_-EtCO_2_ gradient (Pa-_E′_CO_2_) was computed for each patient with the pair of values recorded at the beginning of the NIV session and at 15, 30, 45 and 60 min after the initiation of NIV. The number of Pa-_E′_CO_2_ values of more than ±10 mmHg was reported. The ratio of this number over the total number of measurements represents the proportion of clinically unacceptable EtCO_2_ values. The treshold of 10 mmHg to consider Pa-_E′_CO_2_ as clinically acceptable or not was an arbitrary choice.

All statistical analyses were performed using MedCalc Statistical Software version 12.7.2 (MedCalc Software, Ostend, Belgium). Considering the small number of included patients, non-normal distribution of the results was assumed. All results are given as median [25th and 75th percentile].

The agreement between delta PaCO_2_ and delta EtCO_2_ was assessed by the Bland and Altman method adjusted for the effect of repeated measurements. The differences between each deltaPaCO_2_ and deltaEtCO_2_ values were also computed. The percentage of differences higher than 5 mmHg was reported as they were arbitrarily considered as clinically unacceptable values.

Agreement between PaCO_2_ and EtCO_2_ absolute values was assessed using the Bland and Altman method adjusted for the effects of repeated measurements. Expiratory CO_2_ to PaCO_2_ agreement for values obtained after active and passive complete expirations was also computed with the Bland and Altman method adjusted for the effects of repeated measurements. The gradient between expiratory CO_2_ and PaCO_2_ was computed with the values obtained after active and passive complete expirations respectively. Clinically unacceptable values were arbitrarily defined as values above 10 mmHg. The proportions of clinically unacceptable gradients recorded were compared between normal expiration, active complete expiration, and passive complete expiration by chi-square test. *p* < 0.05 was considered as statistically significant.

## Results

The whole 45-min protocol could be applied to ten patients. In one patient (patient number 4), the NIV treatment had to be interrupted after 45 min because of intolerance. In this patient, the second set of active and passive expiratory manoeuvers was performed after 45 min instead of 1 h, immediately before stopping NIV. Overall, 54-paired data sets of PaCO_2_ and EtCO_2_ from 11 patients (seven men/four women) could be recorded and were included in the analysis. Patients’ demographic and clinical data are given in Table 1. Among the 11 included patients, eight patients had chronic obstructive pulmonary disease (COPD) of various severity (Table 1). Median age was 68 [62 and 77] years old and median SAPS II score was 43 [34 and 44]. Initial blood gas analysis, respiratory rate, inspired fraction of oxygen (FIO_2_), PaO_2_/FIO_2_ ratio, and initial ventilator settings during NIV are mentioned in Table 2.

**Table 1:** **Patient’s characteristics and clinical information.**

**Patient number**	**Sex**	**Age [years]**	**BMI [kg/m** ^**2**^ **]**	**SAPS 2 score**	**Cause of acute respiratory failure**	**Respiratory comorbidity**	**FEV1 (% of predicted value)**	**GOLD classification**
1	F	52	25.3	24	COPD exacerbation	COPD	36	III
2	M	80	22.9	43	Chest trauma with multiple rib fractures	None		
3	M	68	24.5	58	Pneumonia	COPD	43	III
4	M	59	42.6	44	Acute lung injury (bacterial peritonitis)	COPD	57	II
5	M	77	29.3	43	Pneumonia	COPD	32	III
6	M	77	29.4	43	Acute lung injury (pancreatitis)	None		
7	M	63	29.4	31	COPD exacerbation	COPD	33	III
8	M	77	26.1	36	Acute lung injury (peritonitis)	None		
9	F	71	22.0	45	COPD exacerbation	COPD	Not available	Not available
10	F	61	17.2	42	COPD exacerbation	COPD	28	IV
11	F	62	21.5	32	Central hypoventilation (analgesia-sedation)	COPD	54	II
Median		68	25.3	43				
Centile 25		62	22.5	34				
Centile 75		77	29.4	44				

F, female; M, male; FEV1, forced expiratory volume in 1 s; COPD, chronic obstructive pulmonary disease; BMI, body mass index.

**Table 2:** **Respiratory rate, blood gas analysis at inclusion, and main initial ventilator settings**

**Patient number**	**RR [cycles/min]**	**SaO**_**2**_ **[%]**	**pH**	**PaCO**_**2**_ **[mmHg]**	**Bicarbonates [mmol/L]**	**PaO**_**2**_ **[mmHg]**	**FIO**_**2**_	**PaO**_**2**_ **/FIO**_**2**_ **ratio [mmHg]**	**Initial IPAP [cmH**_**2**_ **O]**	**Initial EPAP [cmH**_**2**_ **O]**
1	12	93	7.41	45	27.7	62	0.28	159	15	10
2	17	92	7.38	52	29.6	64	0.35	148	12	7
3	16	88	7.46	45	31.1	53	0.5	89	14	6
4	41	91	7.41	55	34.7	58	0.35	158	12	7
5	21	94	7.32	80	39.8	67	0.4	200	20	8
6	30	99	7.41	55	34.0	112	0.4	138	12	7
7	27	92	7.42	61	38.8	62	0.5	123	8	5
8	29	91	7.40	50	30.7	61	0.5	101	11	6
9	25	90	7.33	58	29.4	58	0.4	145	15	6
10	28	95	7.37	58	32.5	75	0.35	165	12	6
11	20	92	7.47	51	36.7	59	0.3	171	15	6
Median	25	92.	7.41	55.3	32.5	62	0.4	148	12	6
Centile 25	19	91	7.37	51	30.2	58	0.35	131	12	6
Cetile 75	29	93	7.42	58	35.7	65	0.45	162	15	7

RR, respiratory rate; SaO_2_, oxygen saturation in arterial blood; PaCO_2_, carbon dioxide partial pressure in arterial blood; PaO2, oxygen partial pressure in arterial blood gas; PaO_2_/FIO_2_, oxygen partial pressure in arterial blood gas over inspired fraction of oxygen ratio; IPAP, set inspiratory pressure; EPAP, set expiratory pressure.

**Figure 3:**
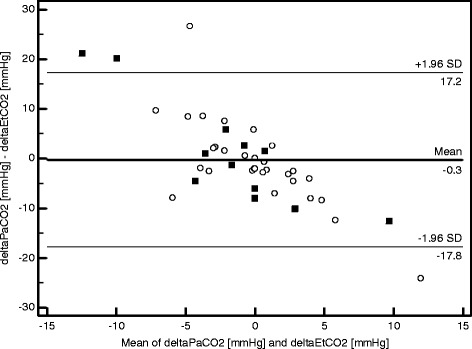
**Bland-Altman plot of agreement between delta PaCO**_**2**_ **and delta EtCO**_**2**_ **.** Bland-Altman plot of agreement between delta PaCO_2_ and delta EtCO_2_. PaCO_2_, CO_2_ partial pressure in arterial blood; EtCO_2_, end-tidal CO_2_; circle markers, COPD patients values; square markers, non-COPD patients values; COPD, chronic obstructive pulmonary disease. The horizontal lines represent the bias and the upper and lower limits of agreement.

During the study period, PaCO_2_ ranged from 39 to 80 mmHg, and EtCO_2_ from 12 to 68 mmHg. At the time of the measurements, delivered inspiratory volume was 724 [597–896] ml and delivered minute ventilation was 18.6 [14.0-22.7] l/min. When assessing the agreement between EtCO_2_ and PaCO_2_ gradients between two consecutive measurements, 43 paired data sets could be analyzed. The bias was −0.3 mmHg and the limits of agreement were −17.8 and +17.2 mmHg. The Bland and Altman graphic representation is displayed in Figure 3. Sixteen of 43 differences (37%) between delta PaCO_2_ and delta EtCO_2_ were higher than 5 mmHg.

**Figure 4:**
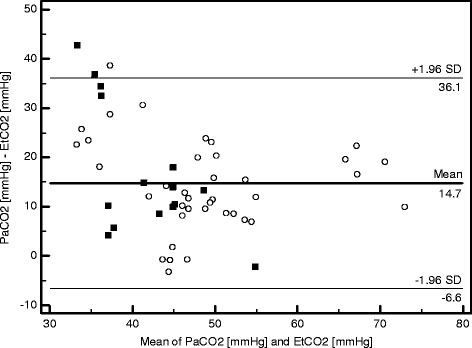
**Bland-Altman plot of agreement between PaCO**_**2**_ **and EtCO**_**2**_ **.** Bland-Altman plot of agreement between PaCO_2_ and EtCO_2_. PaCO_2_, CO_2_ partial pressure in arterial blood; EtCO_2_, end-tidal CO_2_; circle markers, COPD patients values; square markers, non-COPD patients values; COPD, chronic obstructive pulmonary disease. The horizontal lines represent the bias and the upper and lower limits of agreement.

**Figure 5:**
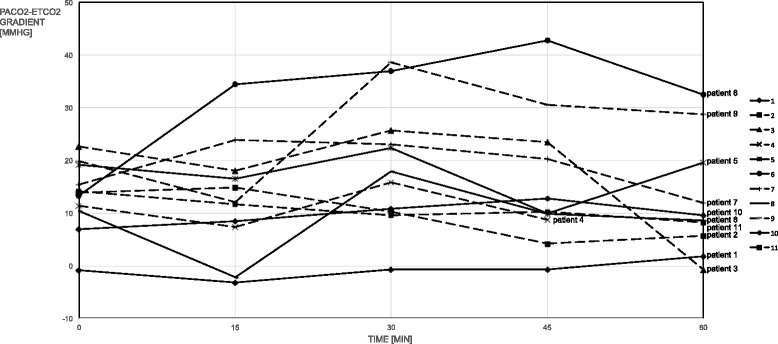
**Evolution over time of PaCO**_**2**_ **-EtCO**_**2**_ **gradient for all the patients.** This figure shows the evolution over time of PaCO_2_-EtCO_2_ gradient for all the patients. PaCO_2_, CO_2_ partial pressure in arterial blood; EtCO_2_, end-tidal CO_2_. Patient numbers 1, 3, 4, 5, 7, 9, 10, and 11 are COPD patients.

When assessing agreement between PaCO_2_ and EtCO_2_ absolute values, bias was 14.7 mmHg and the limits of agreement were −6.6 and 36.1 mmHg (Figure 4). The Bland and Altman graphic representation is displayed in Figure 4 both for COPD patients and non-COPD patients. Pa-_E′_CO_2_ was 12.4 [8.6-20.2] mmHg in median but very high values were documented in some patients (maximal value of 42.7 mmHg) and non-physiologic slightly negative values were observed in one patient (Figure 5). The number of clinically unacceptable values for Pa-_E′_CO_2_ was 35/54 (65%).

**Figure 6:**
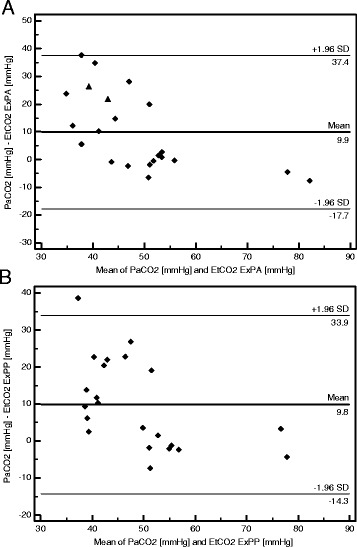
**Bland-Altman plot of agreement between PaCO**_**2**_ **and expired CO**_**2**_ **after active and passive maximal expiration maneuvers. A** Bland-Altman plot of agreement between PaCO_2_ and expired CO_2_ after active maximal expiration maneuver (ExPA). **B** Bland-Altman plot of agreement between PaCO_2_ and Expired CO_2_ after passive maximal expiration maneuver (ExPP). PaCO_2_, CO_2_ partial pressure in arterial blood. In both figures, the horizontal lines represent the bias and the upper and lower limits of agreement.

When we compared agreements between PaCO_2_, concomitant EtCO_2_, and expired CO_2_ after active and passive expiration maneuvers, we had 22-paired data available for each comparison. The bias was respectively 15.7, 9.9, and 9.8 mmHg. Bland-Altmann plots for active and passive expiration maneuvers are displayed in Figure 6A,B respectively. The number of clinically unacceptable gradient values was not different between the three measurements (respectively, 13 (60%), 9 (41%), and 9 (41%), *p* = 0.37).

## Discussion

Our results show that, in patients suffering from hypercapnic acute respiratory failure, measuring EtCO_2_ by a dedicated naso-buccal sensor during NIV was inaccurate to predict either PaCO_2_ variation over time or the absolute PaCO_2_ value. Adding complete passive or active expiratory maneuvers to expiratory CO_2_ measurements did not significantly improve the reliability of PaCO_2_ prediction.

Before discussing the results in more details, we must acknowledge the following limitations of our study. First, only a small number of patients were included. However, a high number of paired EtCO_2_ and PaCO_2_ could be analyzed. As the correlation was poor with very high limits of agreements, it is unlikely that increasing the number of patients would have significantly modified the results. Second, this study used a specific system to measure EtCO_2_ and we cannot exclude that using another device could have yielded different results. Third, only one EtCO_2_ value was recorded at each time. Even if the quality of the corresponding capnogram was carefully checked, we cannot exclude that averaging the values of several respiratory cycles could have provided slightly different results. However, as airway resistance usually not varies between one breath and the following, this effect, if present, should be minor. Fourth, using another patient-ventilator interface or other ventilators, e.g., ICU ventilators equipped with inspiro-expiratory circuits, might also lead to different results. Fifth, during the active and passive complete expiration maneuvers, some patients could potentially not have emptied their lungs enough to reach the residual volume because of maneuver intolerance or because they had to expire through the breathing circuit against the set PEEP. Thus, expired CO_2_ values might not truly reflect expired CO_2_ at residual lung volume. Finally, we cannot exclude that different results could have been found if we had measured EtCO_2_ after stopping NIV treatment. However, as, in clinical practice, it can be difficult or even dangerous to interrupt NIV treatment in patients suffering from acute respiratory failure, we did not test this alternative approach.

EtCO_2_ has been efficiently used for decades in intubated anesthetized patients [7] to monitor PaCO_2_ and ventilation, although many limitations have been recognized, particularly for patients suffering from chronic respiratory diseases (increased VD/VT ratio [8], airflow limitation) or hemodynamic instability leading to ventilation-perfusion mismatches [7,9]. Nasal EtCO_2_ has been successfully used to monitor normocapnic patients with almost healthy lungs undergoing regional anesthesia or recovering from general anesthesia [10]. In line with the results of the present study, two studies performed in spontaneously breathing patients suffering from acute respiratory failure found poor agreement between EtCO_2_ and PaCO_2_ values [11,12]. Oppositely, in more stable and tracheotomized patients, EtCO_2_ values were closer to PaCO_2_ values [13].

In contrast to our results (see Figure 4), in this last study [13], the agreement between EtCO_2_ and PaCO_2_ was better in non-COPD patients than in those suffering from COPD. This last point suggests that during NIV, physiopathological reasons probably do not explain by themselves the poor performances of EtCO_2_ measurement. A possible explanation for the poor agreement we observed between EtCO_2_ and PaCO_2_ during NIV could be the presence of a high airflow and of significant and often variable leaks during NIV that may have caused sampled expiratory gas dilution.

To try to overcome the expected limitation of EtCO_2_ measurement to assess PaCO_2_ absolute values and based on the assumption that, in the absence of major haemodynamic instability and of bronchodilatator administration, Pa-_E′_CO_2_, even if often unpredictable, might be sufficiently constant over an hour in a given patient to enable the tracking of PaCO_2_ evolution, we assessed the time evolution of EtCO_2_ and PaCO_2_. This approach clearly reduced the bias, but the wide limits of agreement preclude its clinical use. Of course, we cannot exclude that physiological reasons, as alveolar recruitment occurring during NIV, could have decreased the VD/VT ratio and contibutated to the poor performance of EtCO_2_ variations to assess PaCO_2_ variations during NIV. However in this situation, EtCO_2_ values would have been closer to PaCO_2_ values at the end of the 1-h NIV treatment, which was not the case.

To try to better assess PaCO_2_, we also attempted to sample gas closer to the alveolar compartment by measuring expiratory CO_2_ at the end of a ‘complete’ expiration (either active or passive) [14] but this approach was also disappointing. Again, this observation contrasts with a study on stable tracheostomized patients [13] and underlines that performing reliable complete expiration maneuvers in acutely ill patients is very difficult.

The present study suggests that other technologies should be considered to non-invasively assess PaCO_2_ and PaCO_2_ over time during NIV. Even if the reliability of using transcutaneous CO_2_ monitoring to assess PaCO_2_ in case of acute respiratory failure is still contoversial [15,16], recent technological improvements in the transcutaneous CO_2_ monitoring technology suggest that this technique could be of interest to monitor PaCO_2_ during NIV. This hypothesis, however, should be formally explored prospectively.

## Conclusions

When a naso-buccal sensor is used, major variations of Pa-_E′_CO_2_ along time and poor limits of agreements between EtCO_2_ and PaCO_2_ preclude the use of EtCO_2_ measurement to predict PaCO_2_ or its variation over time during NIV delivered for acute hypercapnic respiratory failure. Adding complete expiration maneuvers, whether passive or active did not improve PaCO_2_ prediction using EtCO_2_ during NIV. The optimal approach to non-invasively monitor PaCO_2_ during NIV in patients with acute hypercapnic respiratory failure remains to be determined.
